# Human Analogue Safe Haven Effect of the Owner: Behavioural and Heart Rate Response to Stressful Social Stimuli in Dogs

**DOI:** 10.1371/journal.pone.0058475

**Published:** 2013-03-04

**Authors:** Márta Gácsi, Katalin Maros, Sofie Sernkvist, Tamás Faragó, Ádám Miklósi

**Affiliations:** 1 MTA-ELTE Comparative Ethology Research Group, Budapest, Hungary; 2 Department of Agri-Environmental Management, Szent István University. Gödöllő, Hungary; 3 Department of Ethology, Eötvös Loránd University, Budapest, Hungary; Tulane University Medical School, United States of America

## Abstract

The secure base and safe haven effects of the attachment figure are central features of the human attachment theory. Recently, conclusive evidence for human analogue attachment behaviours in dogs has been provided, however, the owner’s security-providing role in danger has not been directly supported. We investigated the relationship between the behavioural and cardiac response in dogs (N = 30) while being approached by a threatening stranger in separation vs. in the presence of the owner, presented in a balanced order. Non-invasive telemetric measures of heart rate (HR) and heart rate variability (HRV) data during the threatening approaches was compared to periods before and after the encounters. Dogs that showed distress vocalisation during separation (N = 18) and that growled or barked at the stranger during the threatening approach (N = 17) were defined as behaviourally reactive in the given situation. While characteristic stress vocalisations were emitted during separations, the absence of the owner did not have an effect on dogs’ mean HR, but significantly increased the HRV. The threatening approach increased dogs’ mean HR, with a parallel decrease in the HRV, particularly in dogs that were behaviourally reactive to the encounter. Importantly, the HR increase was significantly less pronounced when dogs faced the stranger in the presence of the owner. Moreover, the test order, whether the dog encountered the stranger first with or without its owner, also proved important: HR increase associated with the encounter in separation seemed to be attenuated in dogs that faced the stranger first in the presence of their owner. We provided evidence for human analogue safe haven effect of the owner in a potentially dangerous situation. Similarly to parents of infants, owners can provide a buffer against stress in dogs, which can even reduce the effect of a subsequent encounter with the same threatening stimuli later when the owner is not present.

## Introduction

The secure-base and safe haven effects are central features of the human attachment model promoted by Ainsworth [1] and Bowlby [2]. Their attachment theory contains key propositions about the link between attachment and children’s response to threat. The safe haven construct is based on the notion that the attachment figure is serving as a haven of safety to which the child can return in times of threat or distress. Bowlby [3] emphasized the link between attachment and fear, claiming that the availability or unavailability of the attachment figure can serve as a main factor that determines whether a person is alarmed by any potentially dangerous situation. It is argued [4] that human infants evolved with the capacity to use the availability of an attachment figure to calibrate their threat response system at both the behavioural and physiological levels.

Attachment behaviour can only be observed if the attachment system is activated, and at least moderate stress is needed for its activation. The procedure of the Strange Situation Test was designed to evoke increasing stress in human infants by the appearance of a stranger and separation from the mother in an unfamiliar environment. Although a very similar test procedure proved to be effective to observe human analogue attachment behaviour in dog-owner dyads, dogs’ reaction to the strange place and a friendly unfamiliar human was far more diverse than that of human infants [5].

Dog-owner bond is an asymmetrical relationship that is based on dependence mirroring adult-child relationship, thus it can be interpreted in the framework of social attachment [6]. Family dogs’ attachment toward a human is qualitatively different from the relationship with conspecifics. Tuber et al. [7] have found that stress (measured as cortisol level) associated with novel environment can be decreased in dogs by the presence of a familiar human but not by a familiar dog. Recently, several studies have provided evidence for a human analogue individualised attachment relationship between dog and owner (for a review see [8]) applying an adapted version of the Strange Situation Test. Topál et al. [9] provided evidence that even extensive socialization to the human social environment could not provide sufficient conditions for hand-raised wolf pups to develop human-analogue attachment behaviour to the human caregiver. Although convincing evidence for a specific relationship of dogs and their owners fulfilled the operational criteria of attachment [10], the owner’s security providing role has been only indirectly supported [11], [12]. Moreover, for well-socialised pet dogs or assistance dogs [13] this procedure may not be stressful enough to elicit behavioural responses that could explicitly prove the safe haven effect of the owner.

The security providing role of the mother has been investigated in different mammalian species applying both behavioural observations and physiological measures. For example, separation from the mother resulted in significant physiological changes in primates [14], and the infants’ cardiac response indicated stress even when the behavioural reactions to separation could not be observed any more. Somewhat similar results were reported in the case of human infants with insecure-avoidant attachments [15], who did not show protesting behaviour to separation from the mother in an unfamiliar environment, which is typical for securely attached infants, but their elevated heart rate (HR) level indicated their stress in this situation.

Separation from the owner had an increasing effect on adult dog’s mean HR [16], although this phenomenon was most pronounced in the presence of a friendly stranger. When dogs were left alone, their HR decreased [17]. The authors suggested that HR deceleration was a consequence of the animals’ increasing attentional state.

However, the interpretation of changes in mean HR is not simple, because stress-induced and motion-induced changes could overlap [18]. Increased physical activity results in elevated HR in dogs [16]; [19]; [20], so we should be careful when relying on HR as a physiological marker for stress.

Heart rate variability (HRV) is a further cardiac parameter that could be used as a sensitive indicator to assess stress, emotional states and mental activity both in humans (e.g. [21]; [22]) and non-human animals (for a review see: [23]). HRV is a conventionally accepted term to describe variations of instantaneous heart beat intervals (RR intervals) and it reflects the changes in the activity of the autonomic nervous system (e.g. sympatho-vagal balance) of the organism [24]; [25].

Psychological states may have an impact on sympatho-vagal balance, thus resulting in changes in HRV in the absence of measurable alteration in heart rate [23]. For example Zupan et al., [26] found no difference in the HR of tail biter pigs and control (not involved in tail biting) animals in a novel object test; meanwhile the HRV of these two groups differed significantly suggesting an altered regulation of the vagal tone. Tail biters had a suppressed parasympathetic tone in comparison to the controls. Furthermore, learning to control an aversive event was not reflected in HR but was associated with an elevated HRV in sheep [27]. Uncontrollability of the environment, however, was mirrored by decreased HRV suggesting greater sympathetic control over the heart.

In dogs the relationship of behaviour and HRV is less known. Bergamasco et al. [28] found very little correlation between HRV measures and the behavioural data. In a study of Maros et al. [20] the standard deviation of the normal to normal intervals (SDNN), which is an indicator of HRV, was affected neither by posture (laying, sitting, standing) nor by movement (slow walk) of the dogs, but a significant elevation of SDNN during orientation to a favourite toy was reported.

In sum, cardiac activity is considered a useful indicator to evaluate stress in both humans and animals, it thus seems to offer valuable additional information to behavioural observations when we investigate human-analogue phenomena in animals.

In order to model a situation of potential danger in a social context, instead of observing the effect of an approaching friendly stranger (as in the Strange Situation Test), we decided to expose the dogs to a threatening approach, based on the method applied by Vas et al. [29]. In this procedure the approach of an unfamiliar person evoked stress related behaviours, avoidance or moderate aggression from many dogs. This stimulation proved to release consistent and reliable behaviour on the part of the subjects if applied repeatedly: the most behaviourally reactive (threatening) and non-reactive (friendly) dogs tended to show the same set of behaviours in repeated encounters [30]. This procedure seemed plausible to investigate whether physiological effects of stress and the owner’s security providing role in decreasing this stress can be revealed at an unfamiliar place and in a situation when the dogs’ behavioural responses are limited.

In this study we wanted to reveal the relationship between dogs’ behavioural, HR and HRV responses in two social contexts: a) a moderately stressful situation; separation from the owner, b) potential danger; being approached by a threatening stranger in the presence and absence of the owner. In addition, we were looking for physiological evidence in support of one major prediction of the claims about human analogue individual attachment in dogs; that dogs use their owners as a safe haven in stressful situations.

Based on earlier studies on dogs’ individualised attachment relationship, we assumed that dogs’ cardiac activity would change due to the separation from the owner in an unfamiliar environment. Moreover, the change in the HR during separation might depend on whether the dog is really stressed, which can be measured by behavioural variables.

We also hypothesised that dogs’ HR would increase during the threatening approach, but this effect might emerge only in dogs that prove to be sensitive for the threatening approach, that is, in dogs that show relevant vocalisation during the approach.

Finally, we assumed that the presence of the owner provides a buffer for social stress and lessens its effects on the dogs’ HR level and HRV during the threatening social stimulus.

## Methods

### Ethics Statement

All procedures involving dogs and owners were approved by the Ethical Committee of Eötvös Loránd University, and conducted in accordance with the recommendations of the Hungarian State Health and Medical Service (XIV-I-001/520-4). The owners undertook the test on a voluntary basis and they were informed that they would participate in a scientific study.

### Subjects

All dogs (N = 32, 11 females and 21 males, 5 neutered) were living as pets in families. All were large or medium-sized dogs (4 vizslas, 4 labradors, 2 airedale terriers, 2 Belgian groenendaels, 2 border collies, 2 Belgian tervuerens, 1 dogo Argentino, hovawart, kelpie, mudi, wirehaired vizsla, German shepherd, Rhodesian ridgeback and 9 mongrels). The age of the dogs ranged between 1–11.5 years (mean ± SE = 4.7±3.3 years).

We created two groups (N = 16 each) balanced for gender, age and size to control for the two experimental orders (see below).

### Experimental Set Up

The behavioural observations were carried out at the Family Dog Project laboratory (Eötvös Loránd University, Budapest, Hungary) in a 3×5 m experimental room. Three cameras were used to record the behaviour of the dog; one was placed above the door, to show the dog from the front, and two cameras were placed at the left and right side of the room. A fourth camera was behind the dog, pointing at the door to show when the different episodes started and ended (persons entered or left). A fifth camera was used to record only the sounds. An experimenter controlled the events by watching a TV screen that presented the recordings. With a knocking on the window the experimenter could indicate to the owner or stranger when to leave the experimental room.

### Preparation for Heart Rate Recording

Before the start of the experiment the fur of the dog was shaved off in three circles of 5 cm in diameter on the chest. The shaving and fastening of the HR recording equipment on the subject was carried out by the experimenter in a waiting room. Then the owner and the dog were led into the experimental room and the dog could explore for about ten minutes. During this time the experimenter explained to the owner what to do during the episodes. Then the dog was tied by a 1.5-meter long leash to the back wall and the owner sat down on a chair at the wall beside the dog or left the room with the experimenter depending on the test order.

### Procedure

The test included two encounters with the threatening stranger, one in the presence and one in the absence of the owner. In both cases, before and after the encounter the dog was observed in two identical episodes. Two orders were set up to avoid an order effect of the threatening approach. In order A, dogs faced the stranger first in the presence of the owner and later in separation. In the order B dogs were threatened first in separation and then they faced the stranger in the presence of the owner. The threatening approach was enacted by the same unfamiliar female for all subjects.

The episodes of order A were as follows:

### OWNER IS PRESENT

*Episode 1, before threatening* (1 min.): The dog is with the owner. The owner can talk to the dog or touch it to make it calm and still.

*Episode 2, threatening stranger* (1–1.5 min.): The threatening stranger enters the room, steadily gazes at the dog and approaches it very slowly (about 1 short step/10s). The owner is sitting quietly. If the dog is moving or barking continuously, the stranger stops gazing until the dog is quiet or not moving. After one minute the stranger leaves the room. (This episode can last for maximum 90 seconds if the dog has not been still for 15 seconds during the first minute). The experimenter knocks on the window to signal to the stranger when to leave the room.

*Episode 3, after threatening* (1 min.): Same as Episode 1. After one minute the experimenter knocks on the window, the owner tells the dog to stay and leaves the room.

### OWNER IS NOT PRESENT

*Episode 4, before threatening* (3 min.): The dog is in the room alone.

*Episode 5, threatening stranger* (1–1.5 min): The same as Episode 2, with the only exception that the owner is absent.

*Episode 6, after threatening* (3 min): The same as Episode 4.

Order B consisted of the very same episodes but the first and second parts were reversed. In Episodes 1, 2, 3 the dog was in separation, and threatened by the stranger in Episode 2 in the absence of the owner. In Episode 4, 5, 6 the dog was with the owner, and faced the threatening stranger in Episode 5 in the presence of the owner. (In the beginning of episode 4, when the owner came back to the dog we left 1 extra minute for the reunion, and did not include the data of this period into the data set.).

Both sequences were finished by a closing phase when the stranger went to the dog in a friendly manner and petted it. It was important to resolve potential stress and frustration evoked by the test.

As no effect of the 1 minute-long separation on the heart rate level was found in the pilot study, the length of the separation episodes was increased to 3 minutes, assuming that the increased duration would create a more clear context of being separated and therefore evoke more stress in the subjects.

### HR Measure

A holter system (ISAX), developed by Láng and co-workers (e.g. [31]) was used to measure the cardiac activity of dogs. R peaks of the measured ECG signal were detected by using special software of the ISAX then RR intervals (that is all intervals between adjacent QRS complexes resulting from sinus node depolarizations) were measured and stored. After the test, the raw data was transferred to a personal computer. The recording equipment was a small (300 g, 10 cm×5 cm×2 cm) portable plastic box that was placed in a harness on the subject and connected to three ECG electrodes (see details in [19]). The ISAX's built in artefact filtering ensured that the registratum did not contain shorter beat-to-beat interval than 240 ms. This excludes the possibility of the false detection of non R peaks in the ECG signal. Also the ECG recordings were inspected together with the video recordings and sections containing extremely long RR intervals due to the movements of the dog were excluded from the analysis.

The following heart related variables were computed for further statistical analysis: HR (bpm) was derived from RR averages; HR: 60 000/RR, and SDNN (ms) (standard deviation of the normal to normal – also being referred as RR – intervals; 23). The SDNN variable was chosen as one of the simplest variable of the HRV [23].

The behaviour of the dogs was video recorded and analysed after the experiment. The video recording and HR recording was synchronised to the second for each dog with two visible push of an event marker button on the ISAX. Periods where the dogs did not move were used to measure the HR and HRV because we wanted to avoid artefacts caused by movements. This method was already successfully used to evaluate psychological stress in family dogs [20], [32].

### Data Analysis

The video records were analysed by Theme-Coder [33] applying frame-by-frame coding. We coded whining, growling and barking in order to group the dogs into reactive and non-reactive categories regarding their behaviours during the separation episodes and the threatening encounters. Considering that the motor behaviour of dogs was highly restricted in this test, we applied the vocalisation data for the categorisations. Dogs were defined as reactive during separation if they showed high-pitched vocalisation, whining or barking during any of the two separation episodes. For the threatening encounters, dogs that growled or barked during any of the two threatening approaches at the stranger were grouped as reactive individuals. Non-reactive dogs did not growl or bark. (One dog emitted some high pitched bark only in separation orienting at the door not at the stranger. Considering that this dog showed similar behaviour also during the separation before and after the approach, this response was not coded as selective reactivity to the threatening approach.).

We coded body posture in order to control for potential systematic differences during the different episodes.

We coded moving (including intense tail wagging) and changing body posture, because only periods without motor activity (movements, posture change and barking) were considered as periods where only the inner state affected the heart rate. From these periods 15 continuous seconds was considered as the optimal sequences to use for calculating the HR data. In cases where more such periods could be found, the period closest to the end was chosen. If a usable heart rate period was longer than 15 seconds, the heart rate was taken from the middle of the period. The HR data of two dogs (order A) could not be evaluated due to technical problems, thus their data was not included later in the analyses.

Both the HR values and the HRV data followed a normal distribution, so the changes of the dogs’ cardiac responses were analysed with GLM; repeated measures ANOVA. The effects and interactions of three factors were analysed: Owner presence and Threatening encounter (within subject factors) and Order of episodes (between subject factor). We used SPSS 16 for the statistical analyses.

## Results

The dogs were standing, sitting or lying during the episodes, and the distribution of the 3 postures did not differ in the 6 episodes (χ^2^_(10)_ = 15.52; p>0.05), so we did not include this parameter in the further analyses.

### HR and HRV Results of the Whole Sample

First, the effects of three factors on the HR were analysed; Owner presence, Order, and Threatening encounter. No effect of the presence of the owner, and test order was found, that is, HR level of dogs did not change significantly in separation and it was not affected by the order (A or B) in which the dog was tested.

However, significant effect of the threatening approach was revealed, because the HR increased during the threatening encounters. Most importantly, the interaction between owner presence and threatening approach was significant, as the HR increase during the threatening stimulus was more pronounced when the dogs had to face the stranger in separation.

The same analyses were also done for the HRV data. No effect of the test order was found, but both the effect of the owner presence and the threatening approach, and also their interaction were significant. Therefore, the HRV of dogs was affected mainly by two factors and their interaction; separation from the owner increased the HRV when dogs did not face the threatening stranger (**Table 1**).

**Table 1:** Results of the repeated measures ANOVA regarding the change of the cardiac responses of the whole sample of dogs.

	HR (N = 30)			HRV (N = 30)		
	F	df	P	F	df	P
Order (A or B)	0.83	1, 28	0.369	0.17	1, 28	0.682
Owner presence	0.39	1, 28	0.538	11.47	1, 28	**0.002**
Threatening encounter	14.3	2, 56	**0.001**	8.30	2, 56	**0.001**
Owner presence × Threatening	5.75	2, 56	**0.005**	4.13	2, 56	**0.021**
Owner presence × Order	0.90	1, 28	0.350	1.23	1, 28	0.277
Threatening × Order	0.03	2, 56	0.975	0.21	2, 56	0.811
Owner presence × Threatening × Order	2.73	2, 56	0.074	0.75	2, 56	0.477

Significant differences are marked in bold.

### Comparison of the Behaviourally Reactive and Non-reactive Dogs

Considering that well socialised pet dogs may have rather diverse sensitivity for both separation in an unfamiliar place [5] and the threatening approach [30], we also analysed the cardiac responses of the behaviourally reactive dogs.

Out of the 30 subjects, 11 were behaviourally reactive in both contexts; 7 only during separation, 6 only during the threatening encounter, and 6 in neither context.

### Reactivity to Separation

Individuals categorised as reactive whined, barked or did both during any of the separation episodes (N = 18), and non-reactive individuals (N = 12) did not vocalise.

The threatening approach increased the HR in both the reactive and non-reactive group and this effect was attenuated by the owner’s presence in the reactive group (**Table 2**).

**Table 2:** Results regarding the change of the cardiac responses of dogs depending on their behavioural reactivity during **separation** (repeated measures ANOVA).

	HR (N = 30)						HRV (N = 30)					
	REACTIVE (N = 18)			NON-REACTIVE (N = 12)			REACTIVE (N = 18)			NON-REACTIVE (N = 12)		
	F	df	P	F	df	P	F	df	P	F	df	P
Order (A or B)	0.89	1, 16	0.358	0.004	1, 10	0.949	1.96	1, 16	0.18	0.21	1, 10	0.660
Owner presence	0.01	1, 16	0.908	0.15	1, 10	0.707	6.66	1, 16	**0.02**	3.59	1, 10	0.087
Threatening encounter	9.42	2, 32	**0.001**	3.98	2, 20	**0.035**	9.70	2, 32	**0.001**	1.98	2, 20	0.164
Owner × Threatening	5.39	2, 32	**0.010**	1.30	2, 20	0.294	2.96	2, 32	0.066	0.70	2, 20	0.058
Owner × Order	0.04	1, 16	0.853	2.20	1, 10	0.167	0.80	1, 16	0.384	0.11	1, 10	0.752
Threatening × Order	0.02	2, 32	0.876	0.12	2, 20	0.891	0.79	2, 32	0.462	1.58	2, 20	0.230
Owner × Threatening × Order	1.39	2, 32	0.265	1.37	2, 20	0.277	0.63	2, 32	0.537	0.07	2, 20	0.931

Dogs that showed stress vocalisation during separation from the owner were categorised as behaviourally reactive dogs. Significant differences are marked in bold.

**Figure 1:**
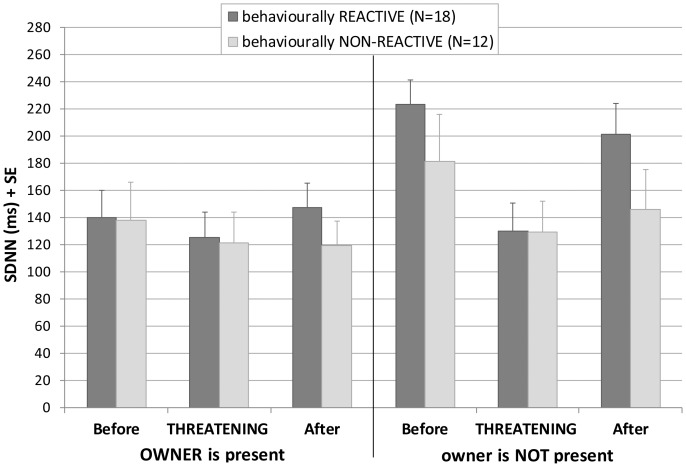
Effects of the threatening approach on the HRV depending on the reactivity to separation. Changes in the mean heart rate variability (SDNN) of behaviourally reactive and non-reactive dogs in the episodes before, during and after the threatening approach of a stranger in the presence and absence of the owner. Dogs that showed stress vocalisation during separation from the owner were categorised as behaviourally reactive dogs.

Both the presence of the owner and the threatening approach had a significant effect on the HRV of the reactive group without any interaction. The HRV of these dogs elevated during separation, but this increase was reduced by the threatening encounter. None of the investigated factors had an effect on the HRV on non-reactive dogs (**Table 2** and **Fig. 1**).

### Reactivity to the Threatening Approach

Individuals categorised as reactive growled, barked or did both during any of the threatening encounters (N = 17). When threatened in separation 8 dogs growled or barked in order A and 9 in order B; when threatened in the presence of the owner 6 dogs vocalised in order A and 7 in order B (all dogs that were reactive in the presence of the owner vocalised also in separation).

The 13 non-reactive dogs’ HR was affected by neither of the three factors nor their interactions. The 17 reactive dogs’ HR was not affected by the order, the presence of the owner or the interaction of these factors. However, during the threatening approach the HR of these dogs increased significantly. This change was independent of the order of the episodes, but we found significant interaction between the owner’s presence and the threatening encounter, and also between the three factors (**Table 3**).

**Table 3:** Results regarding the change of the cardiac responses of dogs depending on their behavioural reactivity during the **threatening approach** (repeated measures ANOVA).

	HR (N = 30)						HRV (N = 30)					
	REACTIVE (N = 17)			NON-REACTIVE (N = 13)			REACTIVE (N = 17)			NON-REACTIVE (N = 13)		
	F	df	P	F	df	P	F	df	P	F	df	P
Order (A or B)	0.12	1, 15	0.736	1.26	1, 11	0.286	0.19	1, 15	0.663	1.30	1, 11	0.278
Owner presence	0.11	1, 15	0.744	0.30	1, 11	0.594	2.27	1, 15	0.153	14.46	1, 11	**0.003**
Threatening encounter	18.31	2, 30	**0.001**	0.95	2, 22	0.403	11.13	2, 30	**0.001**	1.27	2, 22	0.301
Owner × Threatening	5.92	2, 30	**0.007**	2.47	2, 22	0.107	3.49	2, 30	**0.043**	6.72	2, 22	**0.005**
Owner × Order	0.59	1, 15	0.454	0.26	1, 11	0.619	1.05	1, 15	0.321	0.20	1, 11	0.661
Threatening × Order	0.31	2, 30	0.735	3.24	2, 22	0.059	0.95	2, 30	0.399	1.87	2, 22	0.177
Owner × Threatening × Order	4.37	2, 30	**0.022**	0.14	2, 22	0.875	3.13	2, 30	0.058	0.67	2, 22	0.520

Dogs that growled or barked during the threatening encounter were categorised as behaviourally reactive. Significant differences are marked in bold.

**Figure 2:**
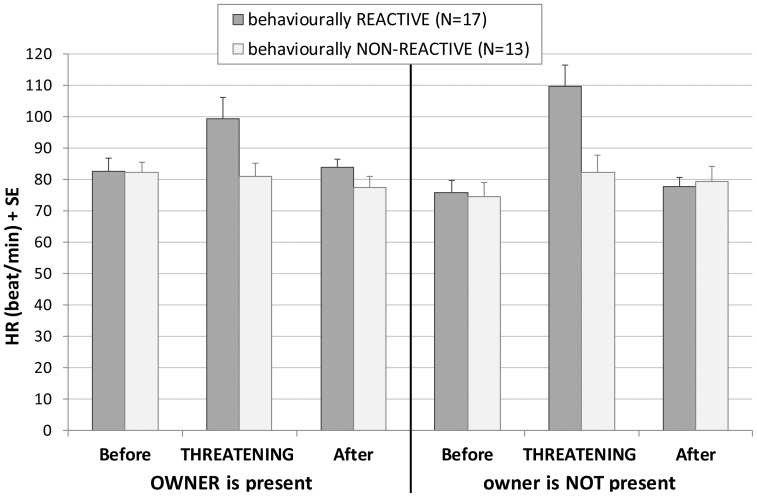
Effects of the threatening approach on the HR depending on the reactivity to the stranger. Changes in the mean heart rate (HR) of behaviourally reactive and non-reactive dogs in the episodes before, during and after the threatening encounter in the presence and absence of the owner. Dogs that growled or barked during the threatening encounter were categorised as behaviourally reactive.

**Figure 3:**
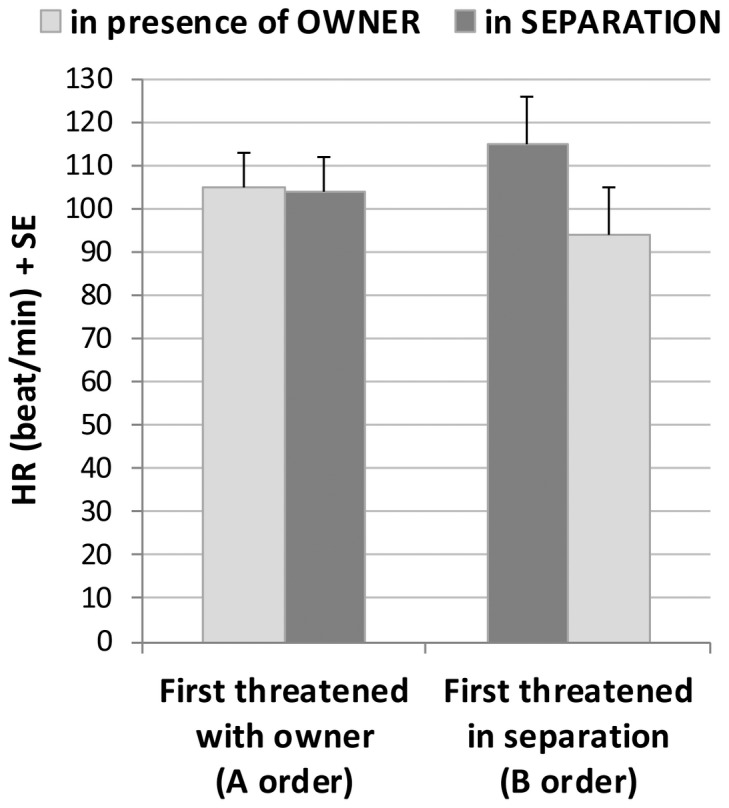
Effects of the test order on the HR in behaviourally reactive dogs. Changes in the mean heart rate (HR) of dogs that were reactive to the stranger in the episodes of the threatening encounter in the presence and absence of the owner in the two orders (A order: N = 8, B order: N = 9).

This means that the HR of dogs increased less when they faced the stranger in the presence of the owner than in separation (**Fig. 2**). Moreover, dogs’ HR increase during the threatening encounter was affected by whether they faced the stranger first in the presence or absence of the owner (**Fig. 3**).

**Figure 4:**
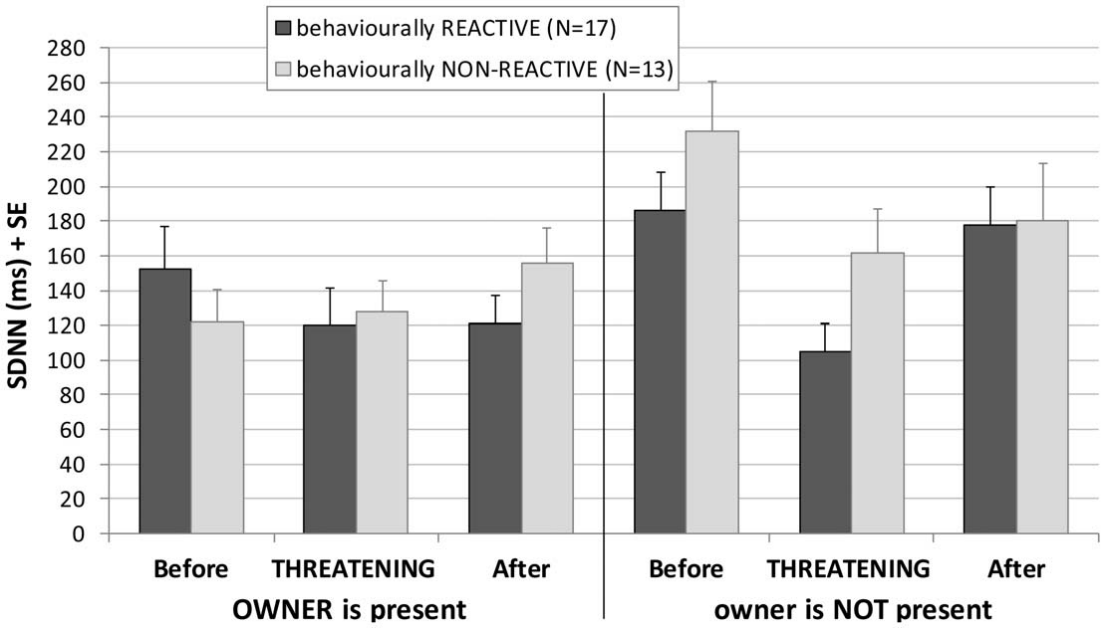
Effects of the threatening approach on the HRV depending on the reactivity to the stranger. Changes in the mean heart rate variability (SDNN) of reactive and non-reactive dogs before, during and after the threatening encounter in the presence and absence of the owner. Dogs that growled or barked during the threatening encounter were categorised as behaviourally reactive.

Reactive dogs’ HRV was affected only by the threatening encounter, not by the order or the owner’s presence. The HRV change was the opposite of the HR, that is, the HRV decreased during the threatening approach of the stranger. Suggested by the interaction of these factors, this decrease was mainly present in the presence of the owner (**Figure 4**).

Non-reactive dogs’ HRV was not affected by the threatening approach, but the separation increased it significantly. There was interaction between the threatening encounter and the owner’s presence, so the effect of the separation was present only when the subjects were not facing the threatening stranger (**Table 3** **)**.

## Discussion

The aim of the present study was twofold. Primarily we investigated whether the dog-owner relationship is analogous to the human infant-parent relationship regarding the security providing role of the owner in potential danger. In addition, we wanted to find cardiac indicators of stress in dogs in two different social situations and reveal their correspondence with behavioural responses.

Previous results reported on dogs’ HR response to separation are controversial, mainly because the change in the inner state is difficult to separate from the parallel change in the activity level. In two studies, applying the modified version of the Strange Situation Test, separation was characterised by pronounced vocalisation and elevated HR despite lower levels of activity [16]; [19]. However, in these test procedures dogs were not only separated from the owner but at the same time also facing a friendly stranger, who tried to initiate interaction (e.g. play) with them. In our study, separation from the owner per se did not seem to have any effect on the HR level of dogs. It is important to note that in our procedure dogs’ motor behaviour was rather limited and standardised across episodes. We suggest that the HR of dogs in separation might be affected by two opposite effects; HR may be increased due to the stress caused by the absence of the owner [16]; [19], but the intense attention towards the potential location of the owner can moderate this effect. Graham and Clifton [34] stated that HR deceleration is a major component of orientation. In our case, separation could evoke stress in the dogs, however, the enhanced attention to the owner (orientation toward the door) might compensate the effect of separation. Maros et al. [20] showed that separation elevated the HR level of dogs only when they were petted by a friendly stranger, but it remained unchanged if the stranger did not try to interact with the dogs during the absence of the owner. Greiveldinger et al. [28] described a similar phenomenon in sheep. An aversive, stressful event (sudden air blast) increased the HR of the animals during an operant learning task, but when they learned to control the aversive stimuli, they showed an increased attention to the task and their HR did not increase anymore. Even though separation had an effect on the HR level only in interaction with the threatening approach, we still found relevant cardiac response to separation, because the HRV was significantly elevated in the absence of the owner. This effect was possibly due to these dogs’ increased orientation towards the presumed location of the owner behind the door. Orientation toward the “favourite ball” also resulted in highly significant HRV acceleration without having much effect on the average HR in dogs [20]. This rapt attention could be broken by the approach of the threatening stranger, which might explain that the HRV returned to the baseline level. The strong influence of the threatening approach on the heart-rate is also indicated by the re-increase of the HRV after the stranger had left and the separation/attention effect re-emerged again.

Variations in the behavioural and physiological reaction to separation from the owner could be explained by differences in socialisation in general and by experience of similar situations (separation from the owner at novel places) in particular. In our study the HRV of dogs highly differed depending on their sensitivity to separation characterised by their stress vocalisation. More detailed analyses revealed that only behaviourally reactive dogs’ HRV increased significantly when their owner left the room, and the threatening stranger had a contrasting effect in both the presence and absence of the owner.

So far HR response in dogs has been measured mainly in the case of encountering a friendly stranger (e.g. [16]) or in non-social situations when the dog was frightened by various stimuli (e.g. [35], [36]). In contrast to the separation from the owner, the threatening approach of a stranger resulted in elevated HR in the whole sample, both when they were threatened in separation and in the presence of the owner. Most importantly, the less HR increase in the presence of the owner during the threatening encounter supported our major hypothesis that the owner is able to provide security for the dog in case of potentially dangerous social situations.

The presence of the owner had an impact also on the HRV change during the threatening encounter, because the HRV decreased significantly (actually returned to the baseline level) only when dogs faced the stranger in the absence of the owner, that is, when HRV was previously elevated by separation. The decrease in the HRV that paralleled the elevated HR during the threatening encounter indicated stress-induced activation due to the potential danger [37].

Our results supported the observations that dogs differ greatly in their sensitivity to the ambivalent social stimuli presented by a slowly approaching and staring stranger [30]. Although our restricted test design largely limited the motor activity of dogs, categorisation of the subjects according to their vocal responses was justified by the corresponding cardiac changes. Interestingly, no HR increase during the threatening encounter could be found in case of behaviourally non-reactive dogs, which means that the reactive sub-group of dogs was responsible for HR increase of the whole sample. According to Graham and Clifton, [34] orientation to novel but not threatening stimuli would decrease HR in contrast to intensive threatening stimuli that would make an increase [20]. Thus we may assume that some non-reactive dogs also experienced stress and showed somewhat elevated HR level, but this effect was counteracted by others that watched the stranger with interest, which could reduce their HR.

We could reveal an additional impact of the owner in case of the behaviourally reactive dogs. Their HR response to the threatening approach depended on whether they faced the stranger first in separation or in the presence of their owner. The owner’s role as a social buffer was supported by the fact, that the same stimuli evoked less increase in dogs’ HR even in separation if they had the possibility to face the stranger first in the presence of the owner.

In reactive dogs, also the HRV change during the threatening encounter was affected by the presence of the owner, which seemed to attenuate the HRV decreasing effect of the stranger. In the non-reactive dogs only the separation effect could be revealed; the HRV increase caused by the absence of the owner indicated intense attention. This main effect was influenced by the threatening encounter, when this focused attention was broken by the appearance of the stranger.

One important lesson from this research is that investigating dogs’ response to various environmental stimulation we should not expect always a strong group level effect, which is based on a unified response. Instead, both the dogs’ behavioural and physiological reactions varies at the individual level (see e.g. [38]; [39]; [40]), which can be explained by either genetic or environmental factors. This means that future studies should take into account the individual variation among dogs that are related to temperament, personality and experiences (e.g. [41]; [42]).

Our results contribute to earlier findings that dogs and humans provide social support for each other in stressful situations [43]; [44]. An important analogy to the human case has been revealed by observing the ability to establish second attachment relationship in shelter dogs [45]; [46] and guide dogs [17]. Such propensity of adult individuals to develop novel attachment relationships has been described so far only in humans. Moreover, under certain conditions such as the loss of the attachment figure (parent or owner), dogs and children may develop analogue behaviour disorders like psychogenic epilepsy, asthma-like conditions, ulcerative colitis, anorexia nervosa (see [47]; [48]).

In this study, we presented direct experimental support for the safe haven effect of owners in case of threat, providing additional evidence for the human analogue nature of the individualised dog-human attachment bond.
